# Recognition, Localization and 3D Geometric Morphology Calculation of Microblind Holes in Complex Backgrounds Based on the Improved YOLOv11 Network and AVC Algorithm

**DOI:** 10.3390/jimaging12030096

**Published:** 2026-02-24

**Authors:** Chengfen Zhang, Dong Xia, Ruizhao Chen, Qunfeng Niu, Tao Wang, Li Wang

**Affiliations:** 1 College of Electrical Engineering, Henan University of Technology, Zhengzhou 450001, China; zhangcf@haut.edu.cn (C.Z.); xiadong9702@163.com (D.X.); ruizhaochen0124@163.com (R.C.); niuqunfeng@haut.edu.cn (Q.N.); 2 Beijing Boshenkang Technology Co., Ltd., Beijing 100102, China; wangtao@tsinghua.org.cn

**Keywords:** microblind holes, multiobject detection, calculation of 3D geometric morphology, improved YOLOv11, AVC

## Abstract

Microblind hole processing quality inspection, especially accurately identifying microblind hole contour features and precisely detecting 3D and morphological parameters, has always been challenging, especially for accurately identifying those of different sizes, depths, and contour features simultaneously. This poses a great challenge for identifying and localizing microblind hole contours based on machine vision and accurately calculating three-dimensional parameters. This study takes cigarette microblind holes (diameter of 0.1–0.2 mm, depth of approximately 35 µm) as the research object. It focuses on solving two major challenges: recognizing and localizing microblind hole contours in complex texture backgrounds and accurately calculating their 3D geometric morphology. An improved YOLOv11s model is proposed for microblind hole image multiobject detection with complex texture backgrounds to extract their features completely. An Area–Volume Computation (AVC) algorithm, which utilizes discrete integral estimation and curve-fitting principles, is also proposed for computing their surface area and volume. The experimental results show that the precision, recall, mAP@0.5, mAP@0.5:0.95, and prediction time of the improved YOLOv11 network are 0.915, 0.948, 0.925, 0.615, and 1.27 ms, respectively. The relative errors (REs) of the surface area and volume calculation of the microblind holes are 5.236% and 3.964%, respectively. The proposed method achieves microblind hole recognition, localization and 3D morphology calculation accuracy, meeting cigarette on-site inspection criteria. Additionally, a reference for detecting other similar objects in complex texture backgrounds and accurately calculating 3D tasks is provided.

## 1. Introduction

In recent years, component miniaturization has been important to the development of micromanufacturing technology, and some progress has been made in dimensional detection equipment [1], which has led to a demand for precise measurement methods for such scales [2]. Accurately detecting miniature parts and microblind holes has attracted attention in various fields, such as aviation, instrumentation, and biomedicine [3,4,5]. In many industries, manufacturing microblind holes is common, but precise measurement of their size and three-dimensional profiles can be expensive or challenging. In particular, the inner contour structure of these microblind holes is often more difficult to measure than the outer contour structure. Despite the challenges involved, precisely measuring the inner contour structure of microblind holes is essential in many fields. With advancements in image processing and laser point cloud scanning technology, accurately detecting microblind holes is significant for improving manufacturing product quality [6] and ensuring production consistency.

Machine vision and deep learning algorithms have been extensively studied in microblind hole recognition and detection. Li et al. [7] proposed a focus measurement operator and an adaptive window fitting method to solve the geometric parameter measurement problem of millimeter-level cooling holes, with a standard deviation (SD) of contour error less than 0.018 mm. Berzal et al. [8] proposed a new method for measuring the volume of arbitrary irregular micropores on rough surfaces. This method was used to calculate the void volume. The model effectiveness was verified by measuring six actual samples. Yuan et al. [9] proposed an improved YOLOv5 network (ClearSight-RS) for remote sensing small target detection. Addressing issues such as complex backgrounds and weak target features, this network enhances the ability to perceive and localize small targets in complex scenarios by means of dynamic convolution and attention mechanisms, thereby providing an algorithmic reference for the recognition of typical small targets in complex backgrounds. Fang et al. [10] proposed a measurement method for microhole profiles in automotive fuel injection nozzles, achieving accurate measurements of microhole diameter and roundness. The experimental results showed that the diameter of the measured microholes was 194.542, and the roundness was 2.551. Yang et al. [11] proposed a new method for measuring the pore size of medicinal glass bottles based on vacuum testing technology. The results show that the proposed method can measure the pore size of medicinal glass bottles, and the indication error of the test results compared to the reference standard value is less than 0.1. Shen et al. [12] proposed a machine vision-based method for detecting microholes in inkjet nozzles. The Canny operator was utilized to extract edge information, which was used to determine parameters such as the aperture size of the microholes. The deviation between the measured aperture size and the nominal aperture size obtained from this method was no more than ±3. Cao et al. [13] proposed an improved two-dimensional detection method for micropores in glass ampoules based on GoogLeNet, achieving a network model accuracy of 99.15% in a self-built dataset.

In the aforementioned microblind hole detection tasks, recognizing and detecting microblind holes and calculating two-dimensional surface contour size ignores three-dimensional morphological information research on microblind holes or only the three-dimensional size information of microblind holes was directly calculated without recognizing and localizing microblind holes. Therefore, applying the abovementioned methods to rapidly detect microblind hole processing quality in practical production fields is difficult. In detecting the quality of microblind holes, laser point cloud scanning, which can provide high-precision three-dimensional contour data of microblind holes, is an ideal method for obtaining accurate and rapid three-dimensional size information under nondestructive conditions. However, in the images obtained after laser scanning conversion, the background texture with microblind holes is complex and variable. Additionally, the contours of the microblind holes are tiny and blurry with various shapes. Accurately recognizing and locating microblind holes with complex textured backgrounds and calculating the geometric three-dimensional morphologies of each microblind hole are difficult. Currently, there is almost no research on the rapid localization of microblind holes with different complex background textures and accurate calculations of three-dimensional hole morphology parameters in practical applications. In addition, high speed is required during the entire detection process efficiently and accurately achieving both the recognition, localization, and precise calculation of geometric three-dimensional morphologies of microblind holes under complex background textures simultaneously poses significant challenges.

With the continuous research on deep learning algorithms, object detection algorithms, which can quickly and accurately identify various objects in images or videos to meet real-time object detection needs, have been widely used in engineering [14,15] and other fields [16,17,18,19]. Chen et al. [20] proposed a new tire detection method with improved YOLOv5, achieving a mean average precision (mAP) of 91.3%. Zhou et al. [21] proposed the DSC-RTDETR model for concrete surface crack detection by improving RTDETR, with YOLOv11 as the backbone, DSConv and dual attention mechanisms integrated, achieving 1.8% higher accuracy and 2.1% higher recall than RTDETR while reducing computational complexity and parameters. Chen et al. [22] proposed an improved single-lens multibox detector (SSD) to achieve rapid vehicle detection in traffic scenes, and the final model achieved mAPs of 82.59% and 84.83% on the BDD100K and KITTI datasets, respectively. Liu et al. [23] proposed an enhanced YOLOv11 for autonomous driving road scene detection with the Roboflow-processed Udacity dataset, achieving 4.6% higher mAP@0.5, 7.6% higher mAP 0.5:0.95, and 6.4% higher recall than the baseline, effectively addressing the detection challenges of distant small targets and dense object clusters. Jiang et al. [24] proposed an improved RetinaNet face detection model called IRNet to detect faces in real-time and achieved detection results of 89.26% and 76.59% on the medium and hard datasets, respectively. Li et al. [25] proposed a real-time detection algorithm for small-object repair parts based on an improved YOLOv11, which improved the feature extraction ability of the network, with mAP@0.5:0.95 and APs@0.5:0.95 increasing by 1.7% and 3.3%, respectively. Aiming at the issues of low detecting precision and the slow processing rate that existed in the traditional target detection methods for aluminum ingot alloy dataset, Kong et al. [15] proposed one for the solder paste similar defects and combined with phase modulation profile measurement technique and improve the YOLOX intelligent detection system. The experimental results show that the best 90.33% detection accuracy is obtained. Guo et al. [26] proposed a small-object detection algorithm based on an improved YOLOv11 to solve problems such as misidentification and missed detection of small targets in object detection, and the mAP for small-object detection reached 94.88%.

This study proposes a comprehensive solution based on an improved YOLOv11 model for microblind hole recognition and detection to meet the demands of rapid identification and positioning of cigarette microblind holes in cigarette quality inspection lines, and the precise calculation of their three-dimensional morphological parameters. as Additionally, an algorithm for three-dimensional morphological parameter calculations to meet the real-time and accuracy requirements for quality inspection lines. This study focuses on the fast and accurate identification and positioning of microblind holes under complex textures and different backgrounds, and the precise calculation of their three-dimensional geometric morphology.

The main contributions of this paper are as follows:

We establish a comprehensive dataset containing 24,398 microblind hole samples across 14 complex texture backgrounds to address the scarcity of specialized training data for micro-scale features. This dataset incorporates diverse hole counts and varying textures to enhance the model’s robustness and generalizability against high-frequency industrial noise. We establish a comprehensive dataset containing 24,398 microblind hole samples across 14 complex texture backgrounds to address the scarcity of specialized training data for micro-scale features. This dataset incorporates diverse hole counts and varying textures to enhance the model’s robustness and generalizability against high-frequency industrial noise.

An improved YOLOv11 multiobject detection network is developed by integrating a VGG13_bn backbone and BiFPN mechanism to ensure precise localization of 0.1–0.2 mm targets. The architecture overcomes the “scale-aliasing” effect in complex backgrounds, achieving a high mAP@0.5 of 0.925 and an inference speed of 1.27 ms to meet real-time industrial requirements.

We propose a novel Area–Volume Computation (AVC) algorithm based on discrete integral estimation and curve-fitting for the precise 3D morphological analysis of irregular holes. This method effectively maintains relative errors below 4% for volume calculation, fulfilling the accuracy demands for on-site quality inspection under non-destructive conditions.

A complete 3D measurement system is integrated to provide a rapid and non-destructive solution for microblind hole quality inspection on cigarette filter tips. This study offers a scalable methodological reference for the automated detection and 3D parameter calculation of similar micro-objects in environments with complex surface textures.

## 2. Data Acquisition and Preprocessing

### 2.1. Data Acquisition

**Figure 1:**
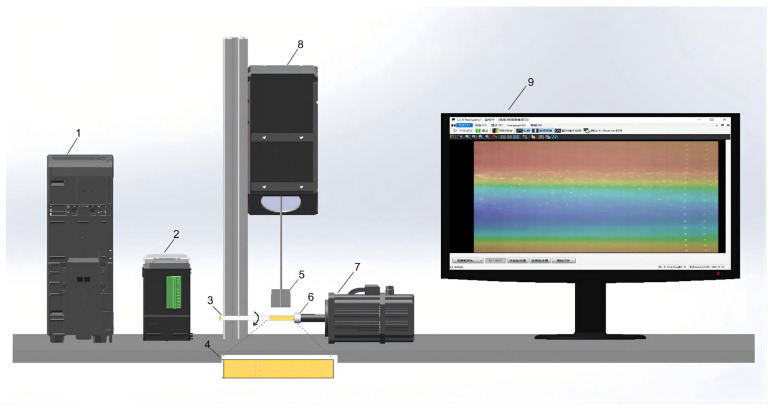
Three-dimensional point cloud data acquisition system: 1 laser scanner controller; 2 servo motor controller; 3 cigarette under test; 4 magnified view of cigarette filter tip; 5 illustration of laser line scanning range; 6 cigarette clamping block; 7 servo motor; 8 three-dimensional contour scanner; 9 screen display.

To ensure model robustness across diverse industrial environments, microblind hole data were collected from 14 cigarette brands (A–N) produced at four different facilities (Factories I–IV). This multi-source sampling strategy introduces a wide variance in material reflectance and production textures, mitigating potential data bias. A specialized 3D acquisition system was developed, featuring a cigarette-rotating laser scanner and an encoder-triggered clamping mechanism (see Figure 1). The system utilized a Keyence LJ-X Navigator to capture high-precision point clouds at a resolution of 3200 × 4096 pixels. For each sample, two synchronized modalities were obtained: brightness point clouds, and height point clouds.

The resulting dataset (detailed in Table 1) comprises 1213 three-dimensional images and 24,398 individual microblind hole instances. To ensure class balance, the number of images per brand was curated between 58 and 99, accounting for the inherent variations in perforation counts (891 to 4136) across different brands. This comprehensive dataset serves as the input for subsequent deep learning models to perform geometric recognition and localization tasks.

### 2.2. Data Preprocessing

**Figure 2:**
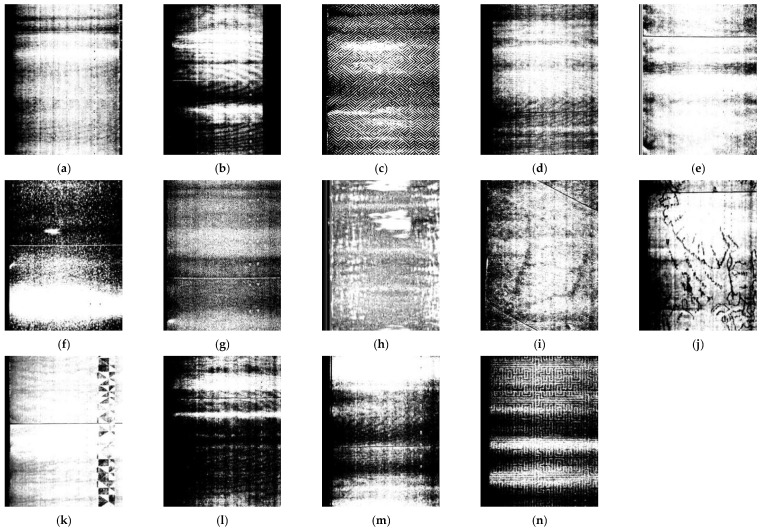
Grayscale images of 14 types of microblind holes on cigarette surface: (**a**–**d**) from Factory I, with diamond-shaped ripple interference; (**e**–**h**) from Factory II, with fine particle interference; (**i**–**l**) from Factory III, with pattern interference; (**m**,**n**) from Factory IV, with zebra-stripe interference.

The problem of locating and recognizing microblind holes was solved from the perspective of point cloud 3D processing to 2D image processing to meet the requirements of rapid detection tasks in real industrial fields. This method enables the rapid location and recognition of microblind holes with complex textural backgrounds. The Matplotlib 3.7.1 algorithm was employed to convert the brightness 3D point cloud data into a 2D image. The 2D image is presented in grayscale mode, which provides a clearer display of the contour details of the microblind holes and reduces the computational complexity of location and recognition to some extent, improving detection efficiency. Due to the influence of the reflective properties of cigarette surface material, the converted grayscale image exhibits complex background texture features. Figure 2 shows grayscale images with a resolution of 2956 × 3784 converted with the brightness data of 14 types of microblind holes, the number of which corresponds to Table 1.

Figure 2 shows that obvious bright and dark stripes exist in the grayscale image, which are caused by the slight deformation of the cigarette due to external forces during clamping. During the rotation of a clamped cigarette, the variation in the distance between the cigarette surface and the laser sensor receiver is one of the main reasons for the appearance of bright stripes in the grayscale image. In addition, the proportion of microblind holes is very small for the entire complex background, and the grayscale images of different types of cigarettes with microblind holes have different background textures. Multiple types of texture features and complex background information are intertwined, which poses a certain difficulty for the tiny microblind hole location and recognition tasks.

Although great efforts have been made to minimize the distortion of cigarette butts and other interferences during point cloud data collection, the grayscale images obtained through the conversion of brightness point cloud data still exhibit noticeable brightness stripes and a significant amount of black and white noise in the background. Therefore, a Gaussian filtering algorithm is proposed based on OpenCV image processing. In setting the Gaussian kernel, a Gaussian kernel that is too large will result in the loss of important features and details of microblind hole contours, while a Gaussian kernel that is too small will not achieve satisfactory smoothing effects on brightness stripes and black and white noise. The Gaussian kernel size is finally optimized to 5, achieving the best smoothing of the brightness stripes and black and white noise.

**Figure 3:**
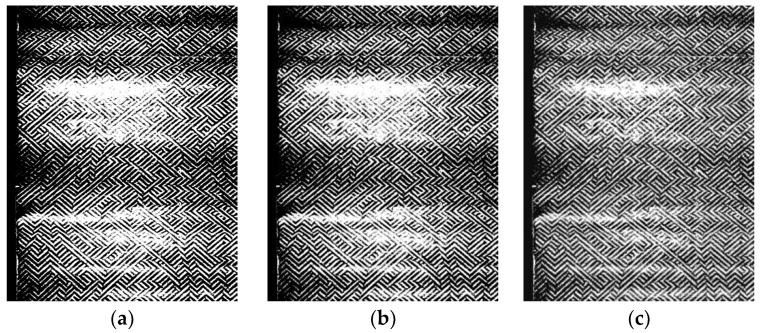
Preprocessing effect comparison: (**a**) Original grayscale image; (**b**) grayscale image after Gaussian filtering; (**c**) grayscale image after contrast enhancement.

Grayscale images smoothed through Gaussian filtering lead to the loss of detailed feature information of microblind hole contours, affecting the recognition accuracy of the network model to a certain extent. The contrast enhancement process method is applied to grayscale images to highlight the details of microblind hole contours. The detail information of microblind hole contours is effectively highlighted while smoothing the brightness stripes and black and white noise in the background of grayscale images by using Gaussian filtering and contrast enhancement. A comparison of the grayscale images before and after pretreatment is shown in Figure 3.

The last preprocessing step is to downsample the grayscale image to transform the resolution from 2956 × 3784 to 1478 × 1892 to significantly increase the inference time and reduce the detection efficiency, while grayscale images with too large of a resolution are input into the network model, which does not meet the demand of real-time detection in the industrial field.

**Figure 4:**
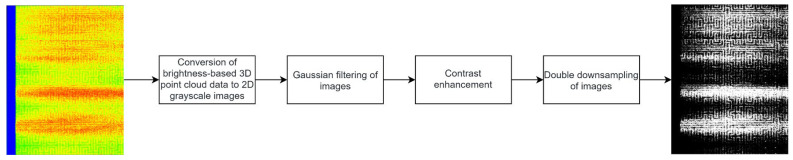
Three-dimensional point cloud data preprocessing flow.

Therefore, total 3D point cloud data preprocessing avoids a large number of computations and accelerates the detection speed under the premise of ensuring model accuracy. The flow of 3D point cloud data preprocessing is illustrated in Figure 4.

**Figure 5:**
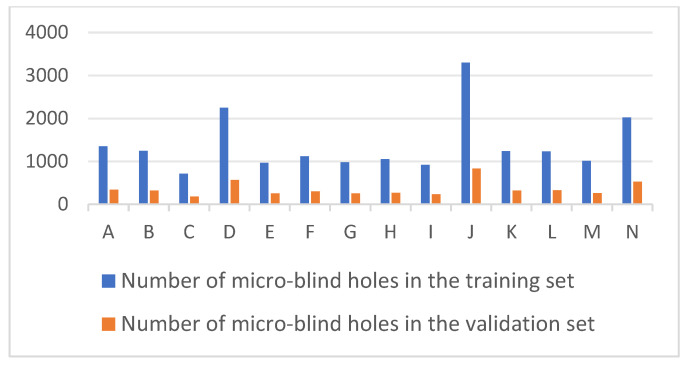
The dataset with 14 types of microblind hole samples.

**Figure 6:**
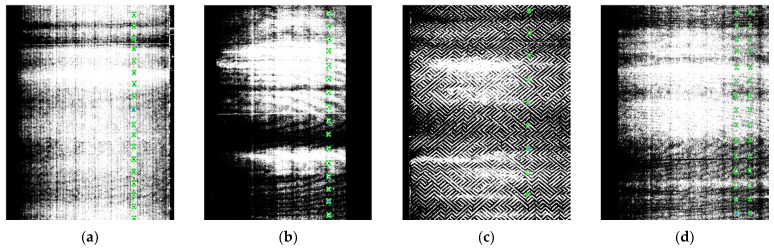
The 5 types of annotated microblind hole images: (**a**–**d**) Annotated microblind hole images from Factory I with diamond-shaped ripple interference.

After preprocessing, the brightness point cloud data were annotated using the image annotation tool labelImg for 14 types of two-dimensional grayscale images with microblind hole contours. Each type of cigarette grayscale image corresponds to a label type. The labels are named consistently with the numbering in Table 1 and stored in the YOLO format. A total of 24,398 microblind hole data points were annotated. The dataset was divided into training and testing sets at an 8:2 ratio. There were 965- and 248-point cloud data points used for the training and testing sets, respectively. The numbers of microblind hole samples were 19,408 and 4990 micro blind hole samples, respectively (see Figure 5). Some of the grayscale images with annotated bounding boxes are shown in Figure 6. The numbers in Figure 5 and Figure 6 are consistent with those in Table 1.

## 3. Proposed Method

### 3.1. Overall Structure of the System

**Figure 7:**
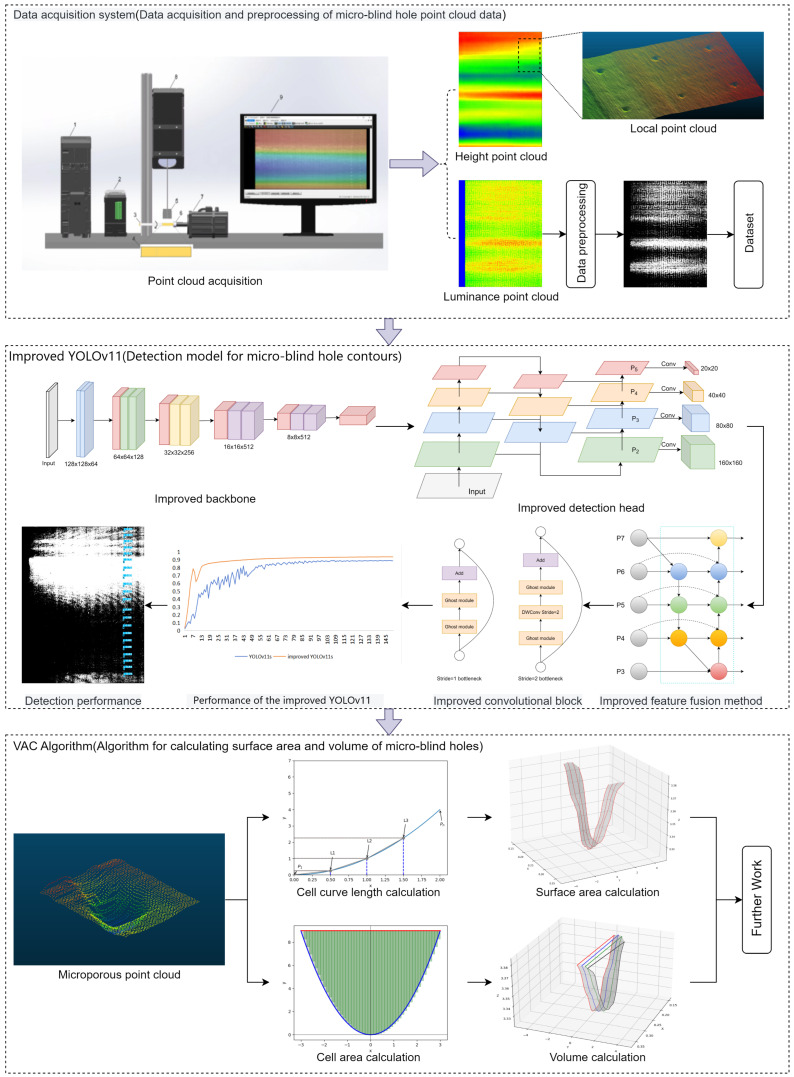
The overall structure of the cigarette microblind hole measurement system.

The designed cigarette microblind hole measurement system is divided into three main parts: point cloud data acquisition, microblind hole location and recognition model, and AVC algorithm. First, a three-dimensional point cloud data acquisition system is constructed to collect three-dimensional point cloud data of different types of microblind hole contours. Image preprocessing is performed on the acquired raw data to ensure that the network model has better performance. Second, an improved YOLOv11 object detection model is developed based on YOLOv11s as the main framework for accurate localization and recognition of microblind holes. The lightweight VGG13_bn serves as the backbone of the network model. A small-object detection layer is introduced, incorporating the BiFPN feature fusion mechanism, while GhostConv and C3Ghost replace Conv and C3k2 in the neck. Finally, a precise AVC algorithm for microblind holes is proposed. Therefore, a new integrated implementation method is proposed for the efficient and accurate precise measurement of the three-dimensional geometric morphology of microblind holes in industrial fields. Figure 7 shows the overall structure of the cigarette microblind hole localization and recognition system and calculation of the three-dimensional geometric morphology.

### 3.2. Implementing the Improved YOLOv11 Model

Single-stage detection network models have recently been developed by optimizing the feature extraction fusion mechanism. Compared with two-stage object detection networks [27], single-stage network models [28,29] are faster in processing object detection tasks and have the characteristics of high detection accuracy and fast detection speed represented by YOLO [30,31,32]. However, in practical cigarette detection tasks, due to the tiny, diverse contours of microblind holes and the complex and variable backgrounds in grayscale image contours, both the detection accuracy and inference speed are still limited. Accurate and complete identification and positioning of microblind hole contours pose great difficulties for the entire detection task. Therefore, a more efficient single-stage object detection network is needed to improve detection performance. In this paper, an improved YOLOv11 microblind hole object detection network is proposed based on the YOLOv11s backbone framework. The rationale behind this specific architectural synergy is dictated by the unique physical constraints of microblind holes (diameter of 0.1–0.2 mm) and the high-frequency noise of cigarette paper textures. Unlike generic object detection, microblind hole recognition requires a delicate balance between fine-grained spatial preservation and rapid semantic convergence. The integration of VGG13_bn, BiFPN, and Ghost-modules is not a mere empirical selection but a targeted design to address the ‘scale-aliasing’ effect where minute hole contours often vanish in deeper feature hierarchies. Specifically:

1. High-performance and lightweight VGG13_bn was used as the backbone of the network model. To fully enhance the ability of the network model to extract microblind hole features, a higher-performance and lightweight VGG13_bn is adopted as the network backbone. The new backbone model adopts a simple structure of convolutional layers and pooling layers, with 13 convolutional layers. Deeper models can obtain multiscale receptive fields at different levels, enabling the capture of higher-level semantic information to fully understand the contour features of microblind holes and solve the problem of locating and identifying various microblind holes.
2. A small target detection layer was added to the neck structure. A detection head specifically for small targets is added on the basis of the three-layer detection heads of the original network model to enhance the ability of the network model to recognize and detect tiny targets. It is composed of shallower feature maps, which have powerful semantic information and precise position information. Compared with the three output layers, the improved network model integrates shallower feature information, ensuring good detection results for small targets while maintaining the lightweight nature of the network model.
3. Optimizing the feature extraction mechanism in the neck structure. The full extraction of shallow and deep feature information is ensured by introducing the BiFPN feature fusion idea and adding cross-scale connections. The fusion of feature information is guaranteed without significantly increasing computational costs. Additionally, the deep separable convolution GhostConv module is introduced to replace regular convolution in the neck structure. C3Ghost replaces the C3k2 module. Without sacrificing accuracy, the complexity of the network model is reduced, greatly optimizing the performance of the network model in detecting microblind holes.

**Figure 8:**
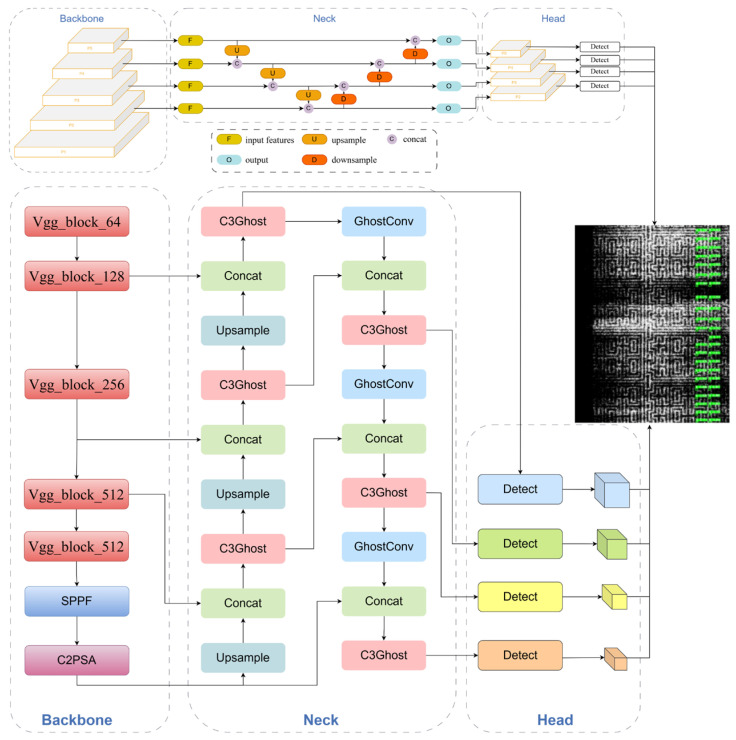
The overall framework of the improved YOLOv11.

The framework structure of the improved YOLOv11 network model is shown in Figure 8 The specific improvements are described in the following three sections.

#### 3.2.1. Improvements to the Backbone Networks

In YOLOv11s, the feature extraction of microblind hole contours in input images is accomplished by the C3k2 module and regular Conv convolution in the backbone network. However, due to the limited local receptive field of the C3k2 convolution block, capturing sufficient global contextual information is difficult. The original YOLOv11s backbone network architecture cannot effectively extract the microblind hole contour features, resulting in inaccurate detection and localization of microblind hole contours in complex backgrounds. Considering that VGGNet [33] has a deeper network structure and can capture a larger range of contextual feature information through multiple consecutive 3 × 3 convolutional layers, its linear, plain-stack structure provides a more consistent receptive field growth. This is mathematically more stable for detecting isotropic features (like circular holes) against anisotropic background noise (like paper stripes). Therefore, the lightweight VGG13bn is adopted as the new backbone of the network model to enhance microblind hole contour feature extraction. VGG13 and VGG16 are two convolutional neural network models based on VGGNet, differing in model depth and parameter quantity. VGG13bn introduces batch normalization (BN) operation into the convolutional blocks based on VGG13, which can accelerate the convergence speed of the network model. Moreover, the BN acts as a spatial frequency filter, effectively suppressing the low-frequency brightness variations shown in Figure 2 while preserving the high-frequency edge information of microblind hole contours. Additionally, the BN normalizes the data distribution, avoiding the vanishing or exploding gradient issues during the training process and effectively improving the stability and generalizability of the model. Compared to VGG16, VGG13bn has a smaller parameter quantity, resulting in faster training and inference speeds. Using VGG13bn as the backbone network for YOLOv11s, although the parameter quantity slightly increases compared to that of the original backbone, the training precision significantly improves, leading to enhanced detection performance of microblind holes by the model.

#### 3.2.2. Improvements in the Detection Head

**Figure 9:**
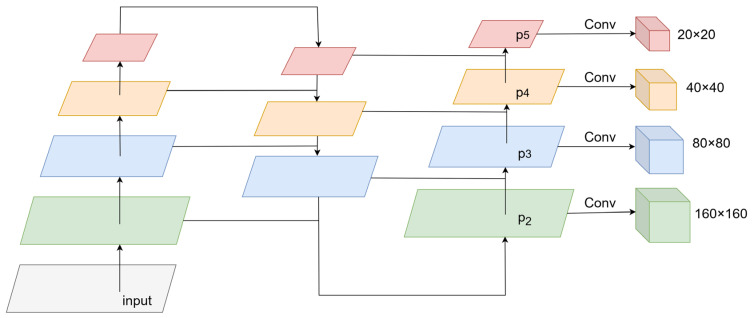
The addition of a small target detection head.

After improving the backbone network, the network model performed five downsampling operations. In the feature maps of three scales (20 × 20, 40 × 40, and 80 × 80) in the original network architecture, large, medium, and small targets were predicted, respectively, which enabled faster extraction of contour feature information for microblind holes in the images. However, because individual microblind hole contour objects have small targets and high resolutions in the entire grayscale image, the P3 layer detection head cannot meet the detection requirements for small target objects. The structure of the original network model YOLOv11s was optimized to enhance the ability of the model to detect small target objects considering the problem of missed detections of small target objects in actual microblind hole identification and positioning tasks. An additional detection head was added to detect small target objects based on the original three detection heads, as shown in Figure 9. The feature map size of the newly added detection head was 160 × 160, which underwent only two downsampling operations in the backbone network and contained abundant shallow position feature information. Each feature pixel corresponded to a receptive field of 4 × 4, which could better extract the feature information of small target objects, enabling the P2 layer to detect small target objects effectively. Fusing deep and shallow feature information was achieved while increasing the detection accuracy introducing the small target detection head, improving the detection capability of small target objects, and ensuring the lightness of the model with little impact on the detection efficiency. “Pi” represents the number of downsampling operations performed on the feature map obtained by the ith layer.

#### 3.2.3. Neck Feature Extraction Improvement

Although backbone networks and detection heads have been continuously improved and optimized, the addition of a small target detection head in the network model incorporates the second layer feature layer, which originally did not participate in feature fusion, into the feature fusion network. Excessive retention of shallow semantic information will lead to severe loss of deep feature semantic information during the training process. Therefore, it is particularly important to preserve relatively deep semantic information on the basis of feature information fusion at shallower levels. An efficient multiscale feature fusion method is needed to further enhance the feature extraction and feature fusion capabilities of the neck structure.

**Figure 10:**
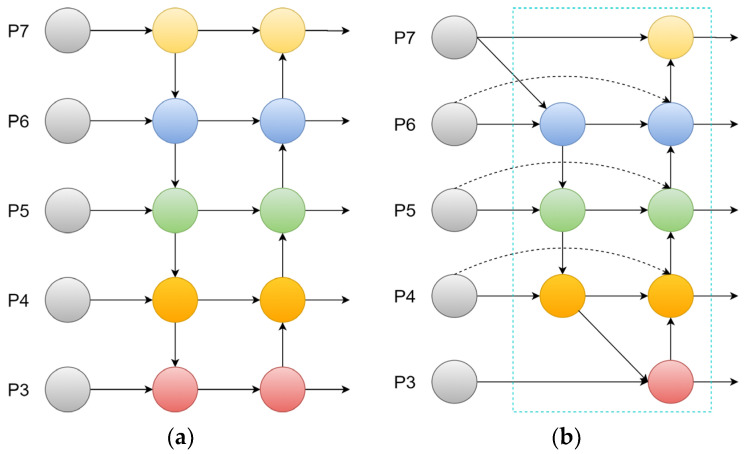
The feature fusion method. (**a**) PANet; (**b**) BiFPN.

An efficient weighted bidirectional feature pyramid network (BiFPN) [34] structure (see Figure 10b) is introduced to improve the neck of the original YOLOv11 network. Since the small target detection head (P2) introduced in Section 3.2.2 significantly increases the feature map resolution (160 × 160), a conventional PANet would incur prohibitive computational costs and risk semantic misalignment. The BiFPN achieves more feature information fusion by shortening the information path between low-level features and high-level features, and its weighted mechanism assigns higher learnable weights to the P2 layer, ensuring that the shallow-seated positioning data is not overwhelmed by deep semantic priors. This improves the detection performance of the network model without adding too many computations.

**Figure 11:**
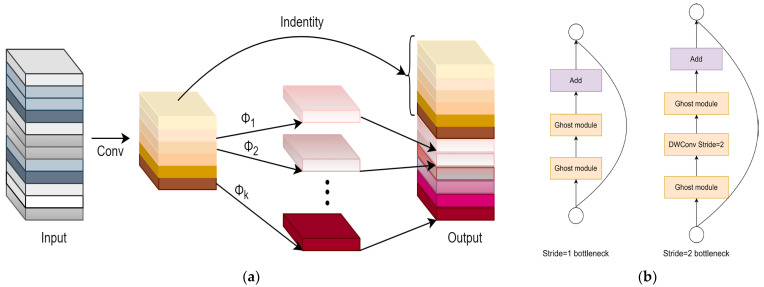
GhostNet. (**a**) GhostModule; (**b**) C3Ghost.

Although the network model improved to a certain extent in terms of detecting microblind holes, in actual application environments, the performance of YOLOv11 network models is easily affected by hardware devices, and excessive parameter quantities are not conducive to the lightweight deployment of network models. To offset the increased complexity of multi-scale fusion and balance detection accuracy and inference speed, the depthwise separable convolution GhostConv module is introduced to replace ordinary convolution in the original network model neck structure, and C3Ghost [35] is used to replace the original C3k2 module (see Figure 11). This combination creates a “structural sparsity” that maintains high inference speed (1.27 ms) without sacrificing the high-dimensional gradient information required to distinguish blurred hole boundaries from background texture, reducing the parameter quantity and computation while ensuring the detection accuracy of microblind holes.

#### 3.2.4. The Algorithm for Calculating the Three-Dimensional Geometric Morphology of Microblind Holes

**Figure 12:**
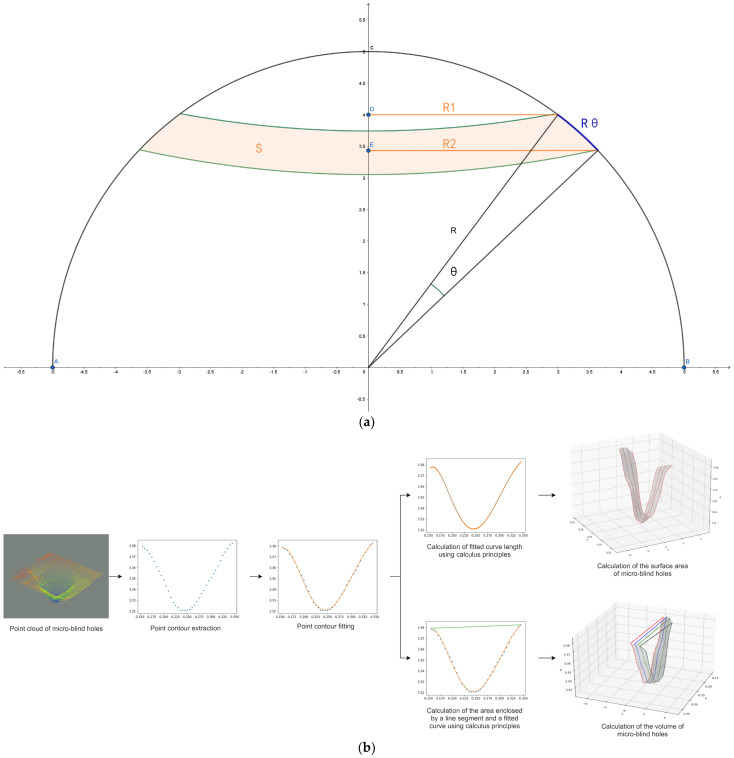
AVC algorithm for geometric quantity calculation. (**a**) Schematic of calculus-based surface area calculation for analogy; (**b**) Workflow for computing the surface area and volume of microblind holes.

The improved YOLOv11 network model enables rapid recognition and detection of microblind hole contours and accurate positioning, laying the foundation for later precise calculations of the three-dimensional geometric morphology of microblind holes. The proposed calculation algorithm maps the obtained coordinate positions of the microblind holes to the three-dimensional point cloud data. Then, the collection of three-dimensional point cloud data and the accurate extraction of the three-dimensional contour point cloud of the microblind holes are performed. Furthermore, the algorithm precisely calculates the contour surface area and volume of the microblind holes. The algorithm for calculating the three-dimensional geometric morphology of microblind holes is named the AVC algorithm, and Figure 12 illustrates the specific implementation process of the AVC algorithm.

Step 1: Mapping of microblind hole contour coordinates and three-dimensional point cloud clipping. The resolution of the grayscale image obtained through the transformation of the brightness three-dimensional data (2956 × 3784) is inconsistent with the size of the three-dimensional point cloud data of microblind hole heights (3200 × 4096). The accurate coordinate positions of the microblind holes obtained through the improved YOLOv11 network model need to be mapped to accurately clip the individual three-dimensional point cloud data of a microblind hole. The calculation formula is as follows:

(1) $$PL\_x=\frac{YOLO\_x\times 3200}{2956},$$

(2) $$PL\_y=\frac{YOLO\_y\times 4096}{3784},$$

where PL_x is the coordinate value in the width direction of the corresponding three-dimensional point cloud of the microblind holes obtained through transformation, PL_y is the coordinate value in the height direction of the corresponding three-dimensional point cloud of the microblind holes obtained through transformation, YOLO_x is the coordinate value in the width direction of the corresponding grayscale image of the microblind holes obtained through the improved YOLOv11 network model, and YOLO_y is the coordinate value in the height direction of the corresponding grayscale image of the microblind holes obtained through the improved YOLOv11 network model.

Step 2: Pixel transformation. Accurately calculating the surface area and volume of microblind holes relies on the accurate measurement of the true distance between two points in the width and height directions of the scanned three-dimensional point cloud data. The characteristics of the laser sensor used in this study fix the distance between two points in the width direction of the three-dimensional point cloud. The number of contour points of the laser beam emitted by the sensor is 3200, and its effective measurement width is 16 mm, from which the true distance between two points in the width direction of the three-dimensional point cloud data can be calculated. However, the true distance between points in the height direction of the three-dimensional point cloud data is not fixed. Since the research object in this study is a cylinder, three-dimensional point cloud data of microblind holes are collected by rotating the cylinder 360 degrees, and the true distance between points in the height direction of the three-dimensional point cloud data is determined by the circumference of the cylinder. The calculation formula is as follows:

(3) $$ST\_y=\frac{Circumference}{4096},$$

In Equation (3), ST_y represents the actual distance in the vertical direction of the three-dimensional point cloud data. Circumference represents the circumferential value of the cylindrical structure with microblind holes.

Step 3: Calculate the contour surface area. The calculus concept is introduced to divide a three-dimensional point cloud of a microblind hole into individual single-point clouds to precisely calculate the contour surface area of irregular microblind holes. Each single-point cloud is then subjected to curve fitting. By calculating the length of each fitted curve and multiplying it by the distance between adjacent fitted curves, the contour surface area of the microblind hole can be accurately obtained. The calculation formula is as follows:

(4) $$Area=\sum_{0}^{n}\left(\sum_{i=1}^{100}\sqrt{{\left({∆x}_{i}\right)}^{2}+{\left({∆y}_{i}\right)}^{2}}\times ST\_y\right),$$

In Equation (4), area represents the contour surface area of the microblind hole, ${∆x}_{i}$ represents the unit distance in the X-direction between adjacent interval points after curve fitting, and ${∆y}_{i}$ represents the unit distance in the Y-direction between adjacent interval points after curve fitting.

Step 4: Volume calculation. Similar to the steps for calculating the contour surface area, the calculus concept is introduced to perform curve fitting on each point cloud. The volume of a differential unit of the microblind hole can be obtained by calculating the area enclosed by the line connecting the start and end points of each point cloud and the fitted curve and multiplying it by the distance between adjacent fitted curves. The accurate volume of the microblind hole can be determined by calculating the volume of each differential unit between adjacent fitted curves. The calculation formula is as follows:

(5) $$Volume=\sum_{0}^{n}\left(\int_{x_{0}}^{x_{1}}(f(x)-g(x))dx\times ST\_y\right),$$

In Equation (5), Volume represents the volume of the microblind holes, $x_{0}$ corresponds to the starting point on a single-point cloud line of the microblind hole, and $x_{1}$ corresponds to the ending point on a single-point cloud line of the microblind hole.

The detailed calculation steps for the contour surface area are as follows. 1. The three-dimensional contour of each microblind hole is composed of n-point cloud lines. The entire three-dimensional point cloud of the microblind hole is divided into individual single-point cloud lines. Curve fitting is performed on each divided point cloud line to obtain the relationship expressed by the fitting curve Equation g(x). 2. The obtained fitting curve is divided into 100 equal parts to ensure that the calculated length of the fitting line is infinitely close to the length of a single-point cloud line. The adjacent interval points after division are connected, and the length of the line segment is calculated using the Pythagorean theorem, which represents the approximate length of a single-point cloud line in a microblind hole. 3. Based on the calculus-based idea of calculating the surface area of a sphere (as shown in Figure 12a), when the angle θ is small enough, R1 is approximately equal to R2, the circular ring area S is approximately equal to R1×R×θ, and R×θ is approximately equal to ST_y. The surface area of the spheres can be approximately calculated by summing all the circular ring areas. For microblind holes, the length of each obtained differential unit of the point cloud line is multiplied by the differential distance between any two of the n-point cloud lines to accurately calculate the contour surface area of the microblind hole (as shown in Figure 12b, which represents the calculation of the contour surface area of the microblind hole after the point cloud is partitioned).

The detailed calculation steps for volume are as follows. 1. The first two steps of the volume calculation are the same as steps 1 and 2 in the calculation steps for the contour surface area. The difference lies in the following steps. 2. By connecting the starting and ending points of a single-point cloud line, the equation representing the line segment corresponding to the upper-end face differential unit in the three-dimensional point cloud of the microblind hole is obtained and expressed by f(x). 3. The area enclosed by the relationship equation f(x) and g(x) is calculated using calculus, which represents a differential unit of the volume of the microblind hole. Then, multiplying it by the differential distance ST_y between any two of the n-point cloud lines gives the volume of the microblind hole (as shown in Figure 12b, which represents the calculation of the volume of the microblind hole after the point cloud is partitioned).

#### 3.2.5. Analytical Modeling of Error Sources in AVC

To ensure the mathematical rigor of the AVC algorithm, the total measurement error $\Delta E_{total}$ can be decomposed into three primary analytical sources: mapping discretization error $\varepsilon_{map}$, curve-fitting residual $\varepsilon_{fit}$, and sensor-induced resolution error $\varepsilon_{res}$. The total error model is defined as:

(6) $$\Delta E_{total}\;=\;\oint_{\Gamma }(\varepsilon_{map}+\varepsilon_{fit}+\varepsilon_{res})d\Gamma ,$$

Mapping Discretization Error ($\varepsilon_{map}$): This arises from the coordinate transformation in Equations (1) and (2). Since the mapping involves non-integer scaling (3200/2956≈1.082), the spatial discretization error is bounded by:

(7) $$\varepsilon_{map}\leq \frac{1}{2}\sqrt{(\Delta PL_{x}{)}^{2}+(\Delta PL_{y}{)}^{2}},$$

Curve-Fitting Residual ($\varepsilon_{fit}$): In Step 3, the error from approximating the point cloud with g(x) depends on the polynomial degree. The residual follows:

(8) $$\varepsilon_{fit}=\int_{x_{0}}^{x_{1}}|P(x)-g(x)|dx,$$

where P(x) is the raw 3D point cloud coordinate.

Integration Approximation Error ($\varepsilon_{int}$): The division of the fitting curve into 100 equal parts introduces a trapezoidal integration error, which is proportional to the second derivative of the contour: $O(h^{2})$, where h is the interval length.

## 4. Results

### 4.1. Implementing the Improved YOLOv11 Model

A 64-bit Windows 11 operating system was used as the experimental platform. The processor used was a 12th Gen Intel(R) Core(TM) i7-12700 CPU @ 2.10 GHz, accompanied by a 64 GB (32 GB × 2) RAM configuration. The system was equipped with an NVIDIA GeForce RTX 3080 graphics card with 10 GB of video memory. The experimental training was conducted within the PyTorch 1.13.1 deep learning framework based on the Python programming language. Python version 3.9 was used, and CUDA version 11.6 was utilized for GPU acceleration. Additionally, the training of the network model involved 150 iterations and 8 batches. The improved YOLOv11 network model was trained using the stochastic gradient descent (SGD) optimizer. After 150 training iterations, the network performance approached a stable state.

Four evaluation metrics for detection performance were adopted, precision (P), recall (R), mAP@0.5, and mAP@0.5:0.95, to evaluate the performance of the proposed improved YOLOv11 network model in terms of microblind hole contours. One evaluation metric for network parameters was adopted as the parameter. mAP@0.5 represents the average detection accuracy of the network model for all detected target categories when the IoU threshold is 0.5. mAP@0.5:0.95 represents the average detection accuracy of the network model under 10 IoU thresholds ranging from 0.5 to 0.95 with a step size of 0.05. The calculation formulas are shown in Equations (6)–(9).

(9) $$P=\frac{TP}{TP+FP},$$

(10) $$R=\frac{TP}{TP+FN},$$

(11) $$AP=\int_{0}^{1}P(R)dR,$$

(12) $$mAP=\frac{1}{n}\sum_{i=1}^{n}{AP}_{i},$$

TP (true positives) represents the number of correctly predicted positive samples, FP (false positives) represents the number of incorrectly predicted positive samples, FN (false negatives) represents the number of incorrectly predicted negative samples, and n represents the number of categories in the self-built microblind hole contour dataset.

Parameters represent the number of parameters in the network model, which is one of the important reference indicators for evaluating the size and performance of the network model.

The surface area (excluding the circular face) and volume of a hemisphere are calculated separately using Equations (10) and (11) to evaluate the accuracy of the proposed AVC algorithm. The relative error (RE), SD, and coefficient of variation (CV) are used as evaluation metrics. The RE represents the relative error between the calculated values and the standard values of the hemisphere’s surface area and volume. SD and CV indicate the standard deviation and coefficient of variation, respectively, which are used to measure the variability in the data.

(13) $$S=\frac{4\pi R^{2}}{2},$$

(14) $$V=\frac{{2\pi R}^{3}}{3},$$

(15) $$RE=\frac{\left|Y-L\right|}{L}\times 100\%,$$

(16) $$SD=\sqrt{\frac{\sum_{i=1}^{n}{\left(X_{i}-\bar{X}\right)}^{2}}{n-1}},$$

(17) $$CV=\frac{SD}{\bar{X}}\times 100\%,$$

In Equations (13)–(17), R represents the radius of the hemisphere, Y denotes the measured value obtained from the AVC algorithm, L represents the calculated value based on Formulas (13) and (14), $X_{i}$ represents the measured value corresponding to an individual sample, $\bar{X}$ is the average measured value obtained from 5 selected samples, and n indicates the number of samples extracted.

### 4.2. Results and Analysis

**Figure 13:**
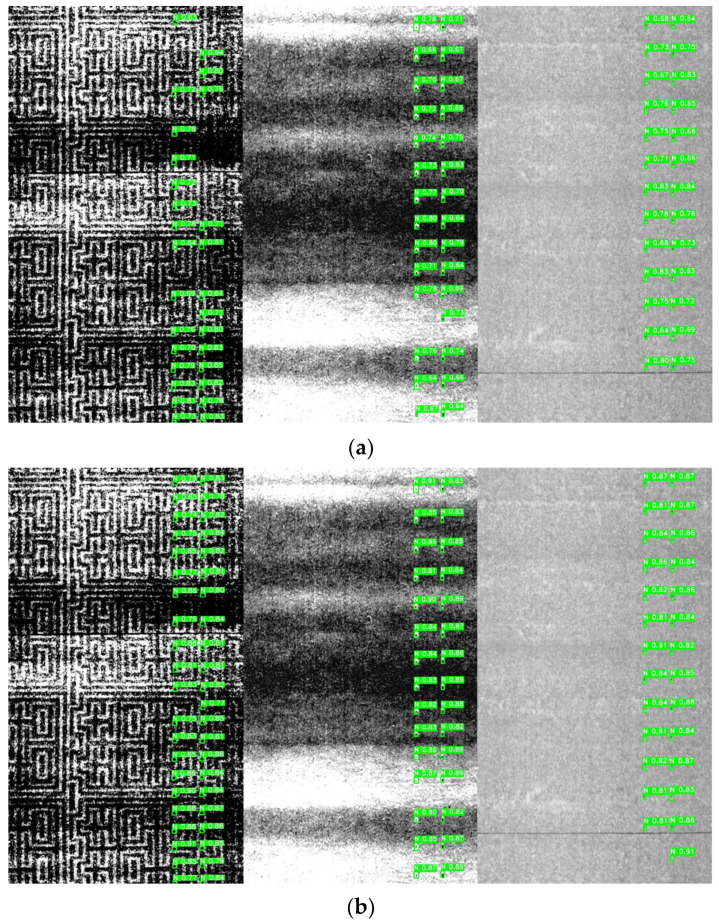
Comparison of the microblind hole detection effect before and after network model improvement. (**a**) YOLOv11s; (**b**) Improved YOLOv11.

YOLOv11s and the improved YOLOv11 models were trained and tested on a self-built dataset of microblind hole profiles for 150 epochs. As shown in Table 2, the detection precision, recall, mAP@0.5, and mAP@0.5:0.95 of the YOLOv11s model were 0.861, 0.893, 0.879 and 0.540, respectively. Compared with YOLOv11s, the improved YOLOv11 model significantly improved the contour detection performance of microblind holes. The improved detection precision, recall, mAP@0.5, and mAP@0.5:0.95 were 0.915, 0.948, 0.925, and 0.615, with increases of 5.4%, 5.5%, 4.6%, and 7.5%, respectively. These results validated the feasibility of the proposed method. Although the improved model had slightly more parameters than YOLOv11s, it was still efficient at recognizing and locating microblind hole profiles and reducing missed or false detection cases, which met the demands of real field detection. Figure 13 shows an example of a detection performance comparison of YOLOv11s and the improved YOLOv11 model.

### 4.3. Ablation Experiment

#### 4.3.1. Validation of Improvements on the Backbone Network

**Figure 14:**
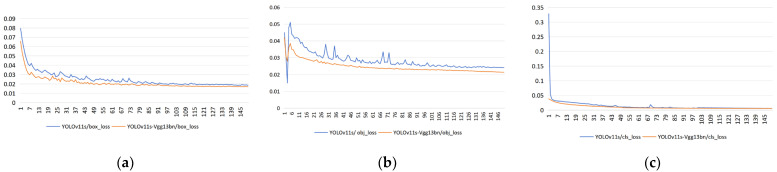
Comparison of model loss before and after improvement. (**a**) Box_loss; (**b**) obj_loss; (**c**) cls_loss.

1. This section validates the feature extraction performance of a new backbone network on microblind hole contours. Ten different network models, including YOLOv11s, EfficientNet, ResNet50, DenseNet121, ShuffleNetV2, MobileNet, VGG11, VGG13, VGG16 and VGG13bn, were compared as backbone networks using a self-built dataset to recognize and locate microblind hole profiles. The experimental results in Table 3 show the following:
2. The EfficientNet, ResNet50, DenseNet121, ShuffleNetV2, MobileNet, and VGG16 models were used as the backbone network for YOLOv11 to extract features of microblind hole profiles. VGG16 and ResNet50, as the backbone networks, achieved similar detection accuracies, but ResNet50 has more parameters and is more complex, which reduces the detection efficiency to some extent. However, VGG16, as the backbone network, has lower parameters while maintaining comparable detection performance, with a precision and mAP@0.5 of 0.895 and 0.912, respectively. Compared to YOLOv11s, there was a significant improvement in the detection precision and mAP@0.5, with increases of 3.4% and 3.3%, respectively; however, the number of network model parameters increased by 129.76%.
3. Comparing YOLOv11s-VGG11, YOLOv11s-VGG13, and YOLOv11s-VGG16, YOLOv11s-VGG13 and YOLOv11s-VGG16 were found to have similar detection accuracies, with differences of only 0.3% and 0.4% in precision and mAP@0.5, respectively. However, the number of parameters of YOLOv11s-VGG13 is reduced by 28.9% compared to that of YOLOv11s-VGG16, making the model more lightweight and improving the detection efficiency to some extent. Although YOLOv11s-VGG11 has the same parameters as YOLOv11s-VGG13, it has lower detection performance and does not meet practical detection needs. Therefore, YOLOv11s-VGG13 performs better overall.
4. Considering that gradient disappearance or explosion may occur during YOLOv11s-VGG13 backbone network training, a BN operation was added to the convolution block of VGG13 to form the YOLOv11s-VGG13bn network structure, which can accelerate the convergence speed of the network model (a comparison of loss functions before and after the improvement is shown in Figure 14.) Compared to YOLOv11s-VGG13, the number of parameters of YOLOv11s-VGG13bn hardly changed; however, all the performance indicators further improved by 0.6%, 0.8%, 0.2%, and 0.4%, respectively. YOLOv11s-VGG13bn achieved the best performance among them.

#### 4.3.2. Validation of Detection Head Improvements

Based on the improvement demonstrated in the previous section, this section validates the model’s performance by introducing a small-object detection head in the neck structure. The effect of the proposed enhancements is verified through comparison.

Table 4 shows that the model with the added small-object detection head significantly enhances the detection performance of microblind holes with tiny contours. This suggests that although the network model parameters are slightly increased with the addition of a high-resolution detection head, the information for tiny objects can be retained, effectively improving the ability of the model to detect and recognize small-object blind hole contours and reducing the problem of missed detection of small objects. The results also demonstrate the effectiveness of the proposed improvements in practical detection tasks and indicate that the position feature information of shallow feature maps has a good auxiliary effect on the detection of small objects. Compared with YOLOv11s-VGG13bn, mAP@0.5 is equivalent, with increases of 0.7%, 1.8%, and 1.3% in precision, recall, and mAP@0.5:0.95, respectively. The proposed model, YOLOv11s-VGG13bn-the P2, is named YOLOv11-VP.

#### 4.3.3. Validation of the Improvements in Neck Feature Extraction

Based on the improvement demonstrated in the previous section, this subsection introduces an efficient BiFPN structure at the neck of the model. The ordinary convolutions are also replaced with the GhostConv module, which utilizes depthwise separable convolutions. The original C3k2 module is replaced with C3Ghost, and the performance of the improved model is validated to compare the advantages of the proposed modifications.

According to Table 5, after introducing the BiFPN structure, the number of parameters remains largely unchanged. Through sufficient feature fusion, the detection precision and mAP@0.5: The 95% CIs of the models are generally more stable than those of the YOLOv11-VP model. The recall and mAP@0.5 slightly improved, and the inference speed also increased. Additionally, adopting GhostConv modules and C3Ghost blocks improved the detection performance of the model for microblind holes. All four performance evaluation metrics improved to varying degrees, with enhancements of 1.0%, 0.2%, 1.2%, and 1.0%, respectively. The final model achieves an inference speed of 1.27 ms and significantly reduces the number of model parameters. The compressed model parameters are reduced by 18.3% compared to the YOLOv11-VP-BiFPN model.

#### 4.3.4. Comparison with Other Detection Methods

To ensure the rigor and comparability of the experiment, this study trained and tested the SSD [29], Faster RCNN [27], YOLOv3 [36], RetinaNet [37], YOLOv11s, YOLOv10b [38], TOOD [39], RT-DETR [40] and improved YOLOv11 models on a self-built microblind hole contour dataset to verify the superiority and effectiveness of the improved YOLOv11 model.

As shown in Table 6, the improved YOLOv11 shows the best overall performance on the self-built dataset compared with all tested models. Compared with YOLOv11s, the proposed improved YOLOv11 model achieves significant improvements in detection precision, recall, mAP@0.5, and mAP@0.5:0.95 for microblind hole contours, with increases of 5.4%, 5.5%, 4.6%, and 7.5%, respectively. Compared with TOOD [39] and RT-DETR [40], the improved YOLOv11 also outperforms them in all evaluation metrics while maintaining a more compact parameter quantity.

In conclusion, the proposed improved YOLOv11 model can efficiently and accurately accomplish the recognition and localization tasks of microblind hole contours in complex backgrounds.

#### 4.3.5. Robustness Analysis Under Industrial Disturbances

To validate the reliability of the proposed system in harsh industrial environments, we conducted robustness tests simulating three common disturbances: illumination fluctuations, mechanical vibrations, and sensor contamination.

**Figure 15:**
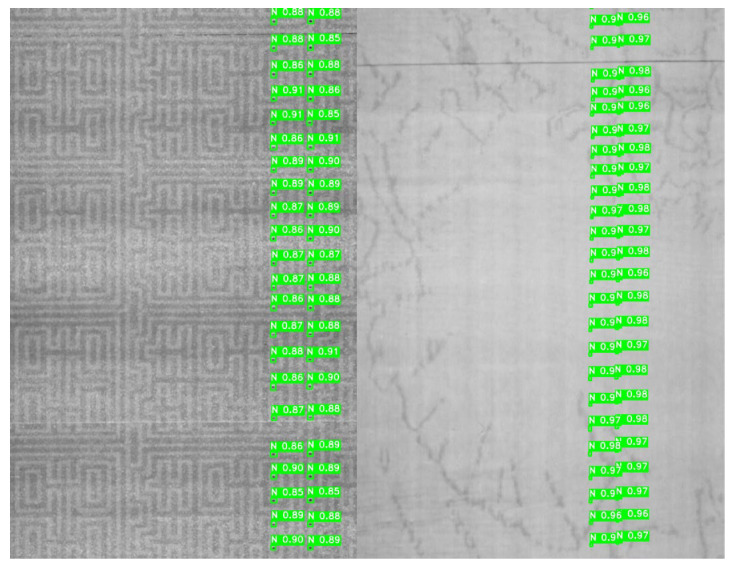
Detection results under illumination fluctuations.

**Figure 16:**
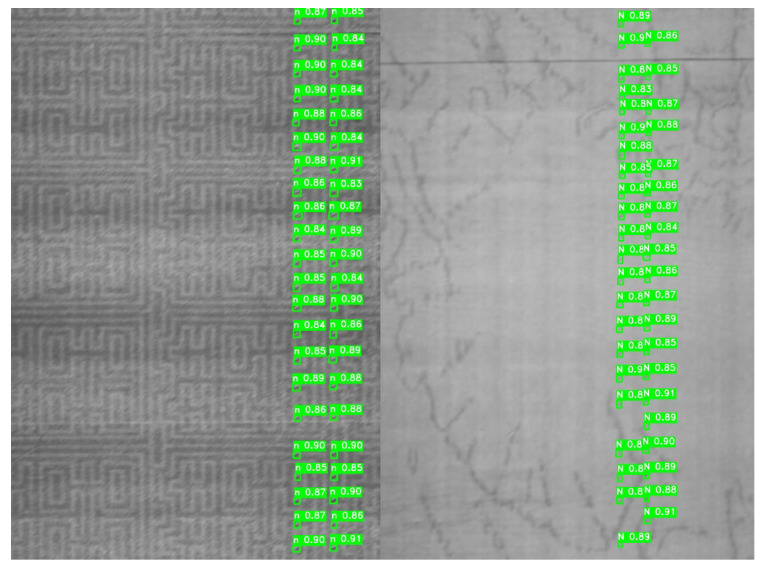
Detection results under mechanical vibration.

1. Illumination Sensitivity: We adjusted the brightness to −30 and applied gamma correction (gamma = 1.5) to simulate non-linear darkening. The improved YOLOv11 maintained a mAP@0.5 of 0.912, showing strong resilience to lighting shifts due to the VGG13_bn’s normalization layers. Typical detection results under illumination variations are shown in Figure 15.

2. Mechanical Vibration: To simulate the jitter of the cigarette clamping mechanism, a motion blur with kernel_size = 20 and near-vertical jitter (angle = 90°) was applied to the testing set. The detection precision only dropped by 2.3%, demonstrating the robustness of the BiFPN feature fusion in preserving structural edges. Typical detection results under mechanical vibration are shown in Figure 16.

### 4.4. Qualitative Analysis of Failure Cases

Despite the significant performance improvements in the improved YOLOv11 model, a qualitative analysis of failure cases reveals specific environmental and geometric constraints that lead to detection inaccuracies. Analysis of the test set identifies three primary error patterns: Boundary Texture Mimicry, where complex background stripes overlap with the circular contours of the microblind holes; Specular Reflection Occlusion, caused by the cigarette’s material properties leading to local overexposure; and Extreme Geometry Distortion, where physical deformation of the cigarette filter alters the projected 2D shape of the holes. As quantified in Table 7, the majority of False Positives (FP) are triggered by High-Contrast Texture Noise, while False Negatives (FN) are predominantly associated with Edge Occlusion. These failure modes suggest that while the network excels in standard complex backgrounds, its sensitivity to non-linear illumination changes remains a secondary challenge for future optimization.

### 4.5. Evaluation of Cross-Factory Generalization

A critical requirement for industrial deployment is the model’s ability to generalize across different factories. We conducted a cross-validation experiment using a “leave-one-factory-out” strategy. The model was trained on data from Factories I, II, and III, and subsequently tested on unseen samples from Factory IV.

As shown in Table 8, the model achieved a mAP@0.5 of 0.896 on the unseen factory data, which is only a marginal decrease compared to the intra-factory testing results (0.925). The AVC algorithm also maintained a volume RE below 4.5% across different sites. This high generalization performance is attributed to the GhostConv-based neck architecture, which focuses on universal morphological features rather than overfitting to specific factory-dependent background textures. The results confirm that the proposed framework can be rapidly deployed across various manufacturing sites without extensive retraining.

### 4.6. Cross-Domain Generalization on Industrial Micro-Defects

**Figure 17:**
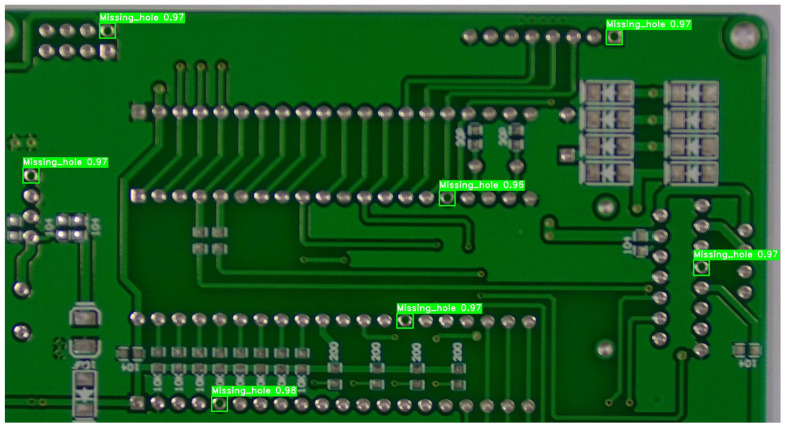
Visual detection results of missing_hole defects on PCB boards from the HRIPCB dataset.

To address the concern regarding the potential domain-specificity of the proposed model, a cross-domain generalization experiment was conducted. We evaluated the architecture on the HRIPCB dataset (released by the Intelligent Robot Open Laboratory, Peking University), focusing on the “missing_hole” defect category. Unlike cigarette filters, PCB surfaces exhibit rigid geometric patterns and diverse metallic reflections, providing a rigorous test for the model’s spatial feature invariance. The detailed recognition results are illustrated in Figure 17.

As shown in Table 9, the improved YOLOv11 model demonstrates superior transferability compared to the baseline models previously discussed in Section 4.3.4. On the HRIPCB dataset, our model achieved a Precision of 0.987, a Recall of 0.976, and a mAP@0.5 of 0.982. This represents a significant performance leap over the standard YOLOv11s (Precision: 0.942) and Faster R-CNN (Precision: 0.915).

The exceptional precision (>98.5%) on the HRIPCB dataset is attributed to two factors:

1. The P2 High-Resolution Head: The 160 × 160 feature map is particularly sensitive to the sharp circular edges of missing drill holes on PCB boards.
2. BiFPN and Ghost-Modules: These ensure that the model captures the high-frequency contrast between the substrate and the missing hole without being distracted by the complex routing patterns (traces) of the PCB.

The results empirically confirm that the proposed framework is not merely a specialized tool for cigarette micro-blind holes but a robust solution for diverse industrial micro-scale feature detection.

### 4.7. Metrological Validation of the AVC Algorithm Using Certified Standards

**Figure 18:**
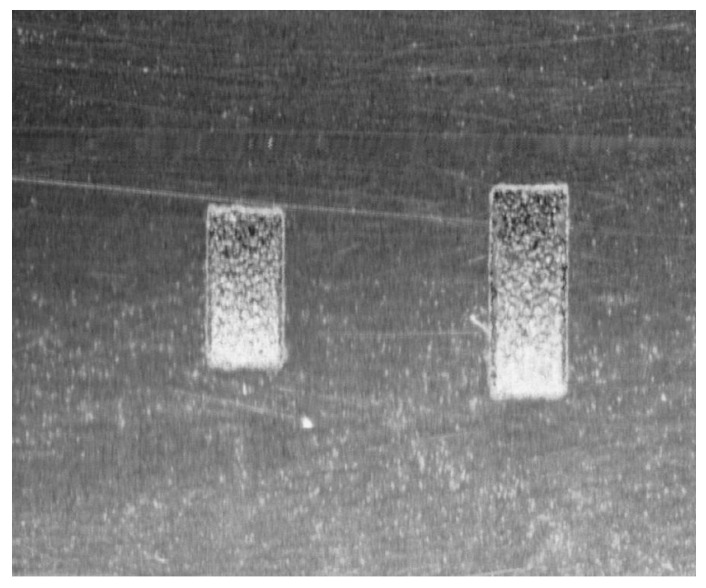
Intensity map of the certified microblind hole standards.

To address the requirement for independent metrological validation, the proposed AVC algorithm was tested against two high-precision certified microblind hole standards. These standards feature rectangular geometries, which allow for a more rigorous evaluation of the algorithm’s geometric invariance compared to simple hemispherical models. The intensity map of the certified standards is shown in Figure 18.

The dimensions of the standards are:

1. Standard S1: Depth 0.1 ± 0.005 mm, Width 1.0 ± 0.005 mm, Length 2.0 ± 0.005 mm (Theoretical Volume: 0.200 mm^3^; Inner Surface Area: 2.600 mm^2^).
2. Standard S2: Depth 0.2 ± 0.005 mm, Width 1.0 ± 0.005 mm, Length 2.8 ± 0.005 mm (Theoretical Volume: 0.300 mm^3^; Inner Surface Area: 3.800 mm^2^).

The measurement results are summarized in Table 10. The relative errors (RE) for volume calculation were 3.45% for S1 and 3.79% for S2. These results demonstrate that the AVC algorithm maintains high precision across different scales and geometric profiles, effectively bridging the gap between computer vision localization and industrial metrological standards.

### 4.8. Ablation Study and Sensitivity Analysis of the AVC Algorithm

To address the individual contribution of each component within the AVC algorithm and validate the theoretical error model proposed in Section 3.2.5, a systematic ablation study was conducted. We evaluated three key factors: the polynomial degree for curve fitting (g(x)), the discrete integration resolution (N divisions), and the mapping scale precision. The analysis was performed using the certified standard S1 as the benchmark.

#### 4.8.1. Effect of Curve-Fitting Order ($\epsilon_{fit}$)

The curve-fitting component is designed to smooth the discrete point cloud and minimize sensor noise. We compared the performance of linear, quadratic, and cubic (current) fitting models. As shown in Table 8, linear fitting (Degree 1) fails to capture the subtle curvature of the microblind hole bottom, resulting in a significantly higher RE for volume (8.42%). Transitioning to cubic fitting (Degree 3) reduced the RE to 3.45%, confirming that higher-order polynomials effectively minimize the residual $\epsilon_{fit}$ as modeled in Section 3.2.5.

#### 4.8.2. Impact of Discrete Integration Step Size ($\epsilon_{int}$)

The integration approximation error $\epsilon_{int}$ is theoretically $O(h^{2})$. We varied the number of equal parts (N) for the fitting curve from 20 to 200. The results in Table 8 indicate that while N=20 leads to a coarse approximation with 7.15% RE, increasing N to 100 provides an optimal balance between accuracy (3.45% RE) and computational efficiency (latency < 52 ms). Further increasing N to 200 yielded negligible improvements (3.41% RE) but increased processing time by 45%.

#### 4.8.3. Sensitivity to Mapping Precision ($\epsilon_{map}$)

To evaluate the impact of the non-integer scaling mapping defined in Equations (1) and (2), we compared the proposed adaptive mapping with a fixed-integer rounding approach. The adaptive mapping reduced the surface area RE by 1.2% by preserving sub-pixel spatial relationships, validating the necessity of the precision mapping component.

The results in Table 11 empirically demonstrate that the cubic fitting and the N=100 integration step are the most critical components for maintaining high geometric accuracy. This experimental evidence aligns with the mathematical error bounds $\epsilon_{fit}$ and $\epsilon_{int}$ established in the analytical modeling section, proving the robustness of the AVC framework for micro-scale morphology.

### 4.9. Comparative Analysis of AVC and Classical Mesh-Based Baselines

To further elucidate the novelty and superiority of the AVC algorithm, a comparative study was conducted against the standard Mesh-based Integration (MI) method [41], which is a common baseline in 3D reconstruction. The MI method involves generating a water-tight mesh via Delaunay triangulation and summing the signed volumes of the tetrahedra. Both algorithms were tested using the S1 Certified Standard (Theoretical Volume: 0.200 mm^3^; Surface Area: 2.600 mm^2^) under identical hardware conditions.

As summarized in Table 12, the AVC algorithm significantly outperformed the MI method in both accuracy and efficiency. While the MI method is highly dependent on the raw density and local quality of the point cloud, often leading to overestimation due to high-frequency surface noise (Relative Error of 7.24% for volume), the AVC algorithm achieves a lower volume RE of 3.45% through its cubic-fitting smoothing mechanism.

The results demonstrate that the AVC algorithm provides a superior balance between metrological precision and computational overhead, particularly for micro-scale objects where raw point cloud data may contain significant scanning artifacts.

### 4.10. Sensitivity Analysis and Error Propagation from Localization to 3D Quantification

To establish a rigorous methodological link between the upstream object detection and the downstream geometric calculation, a sensitivity analysis was performed to evaluate how localization inaccuracies propagate to the final surface area and volume estimations. In the proposed framework, the AVC algorithm utilizes the bounding box coordinates generated by the improved YOLOv11 as the spatial cropping window for 3D point cloud extraction. Consequently, a spatial jitter in the bounding box (defined by center coordinates x,y and dimensions w,h) directly alters the integration domain Ω for Equations (4) and (5).

To quantify this effect, we manually introduced controlled pixel-level perturbations to the optimal bounding boxes on a representative subset of the testing data. The perturbation magnitude ranged from ±2 to ±15 pixels, simulating the common localization fluctuations observed in complex backgrounds. The experimental results, as summarized in Table 13, illustrate the correlation between Intersection over Union (IoU) degradation and the resultant Relative Error (RE) of 3D parameters. It is observed that the AVC algorithm exhibits a high degree of mathematical robustness when the localization offset is within a reasonable industrial tolerance. Specifically, a localization shift of ±5 pixels—which corresponds to a decrease in IoU to approximately 0.92—only leads to a marginal increase in the RE of surface area (from 5.236% to 5.612%) and volume (from 3.964% to 4.285%). This stability is attributed to the fact that the microblind hole contours are centrally distributed within the detected boxes, and the calculus-based curve fitting (CCF) within the AVC algorithm effectively smooths minor boundary noise introduced by slight window misalignments.

However, as the localization offset exceeds ±15 pixels (IoU < 0.8), the RE for volume calculation increases more sharply compared to the surface area. This phenomenon occurs because large spatial shifts cause the cropping window to truncate the deepest part of the hole or include significant background noise, thereby distorting the integral ∫(f(x)−g(x))dx. Nevertheless, given that the improved YOLOv11 achieves a high mAP@0.5 of 0.925 and precise localization, the operational fluctuations remain well within the robust regime of the AVC algorithm. This error propagation analysis confirms that the proposed detection-to-calculation pipeline is not only accurate in its individual components but also highly resilient as an integrated system for industrial inspection.

### 4.11. Industrial Real-Time Performance and Latency Analysis

To further validate the “real-time” claims for industrial deployment, a comprehensive system-level latency analysis was conducted. The total processing time ($T_{total}$) for a single inspection cycle is decomposed into four stages: data acquisition ($t_{acq}$), image preprocessing ($t_{pre}$), YOLOv11 detection and localization ($t_{det}$), and AVC-based 3D morphology calculation ($t_{avc}$).

As shown in Table 14, the end-to-end latency on the primary testing platform (RTX 3080) is approximately 346.67 ms per image, which translates to an inspection speed of about 2.9 frames per second (FPS). The AVC algorithm, despite its calculus-based integration, maintains a relatively efficient latency of 51.4 ms due to the optimized curve-fitting logic.

Furthermore, to assess hardware sensitivity, the model was tested on a mid-range industrial embedded platform (NVIDIA Jetson AGX Orin). The total latency increased to 637.15 ms (corresponding to ~1.6 FPS), which still meets the basic industrial inspection criteria for low-to-medium speed production scenarios, demonstrating the robust deployment potential of the proposed framework across varying hardware scales.

## 5. Conclusions

In practical three-dimensional morphology of cigarette microblind hole detection tasks, efficiently recognizing and locating the contours of different microblind holes in complex backgrounds and accurately measuring their surface area and volume is difficult. This paper takes cigarette microblind holes (diameter of 0.1–0.2 mm, depth of approximately 35 µm) as the research object and focuses on solving two major challenges: the recognition and localization of microblind hole contours in complex texture backgrounds and the accurate calculation of the 3D geometric morphology of microblind holes. An improved YOLOv11 network model is proposed to recognize and locate the contours of different types of microblind holes. An AVC algorithm is also proposed to further calculate the surface area and volume of microblind holes in a three-dimensional morphology. The proposed detection method meets the actual detection requirements of industrial sites. This study achieves the following innovations:

1. A three-dimensional point cloud data acquisition platform for microblind holes was established, and 3D perforation data of 14 different cigarette brands with different perforation amounts ranging from 9 to 44 and different complex texture backgrounds were collected. A data preprocessing algorithm is researched to reduce the amount of parameter computation calculations. Considering the diversity of microblind hole contours and the complexity of backgrounds, 1213-point clouds were collected to establish a dataset of 24,398 microblind hole contour samples to better meet the microblind hole detection task and improve the model’s generalizability.
2. We propose an improved YOLOv11 network model with a new backbone, an improved feature fusion mode and added detection heads. VGG13bn serves as its new backbone network, and the extracted deep-level features can capture higher-level semantic information, significantly improving the microblind hole detection performance. A small target detection layer is introduced to improve the detection performance of smaller microblind hole contours without significantly increasing the model parameters. Moreover, to strengthen the feature extraction and fusion of the neck structure, a BiFPN structure is introduced to fuse more feature information without significantly increasing the model’s parameter quantity. Finally, the GhostConv and C3Ghost modules are introduced in the neck structure to replace the Conv and C3k2 modules, respectively, reducing the model’s parameter quantity while improving its detection precisionand efficiency. The improved YOLOv11 network model performs best at efficiently completing microblind hole contour recognition and location tasks under complex background textures.
3. The AVC algorithm was used to efficiently and accurately calculate the surface area and volume of irregular microblind holes. This approach solves the problem of high-precision measurements of irregular 3D microblind hole morphologies and meets the measurement requirements of industrial sites.

The detection precision, recall, mAP@0.5, mAP@0.5:0.95, parameter quantity, and detection time of the proposed improved YOLOv11 network model are 0.915, 0.948, 0.925, 0.615, 19,250,030, and 1.27 ms, respectively, ensuring detection precision and efficiency under the premise of not having a large number of model calculation parameters. The performance of the proposed method was validated, making it highly applicable to industrial sites.

However, this study has certain limitations. For microblind hole contours with overly complex backgrounds, the model may have a small number of missed detections, and the calculation accuracy of the AVC algorithm needs further improvement. Subsequent work should consider the following aspects:

1. Further optimizing the detection performance of the network model for detecting microblind hole contours in complex backgrounds with various textures and stripes.
2. The AVC algorithm is optimized to minimize measurement errors and ensure computational efficiency for microblind holes with poor regularity in 3D contour morphology.

## Figures and Tables

**Table 1:** Source of the microblind hole datasets.

Serial Number	Facility	Number of Point Clouds	Number of Microblind Holes
A	I	94	1692
B	I	98	1568
C	I	99	891
D	I	94	2820
E	II	87	1218
F	II	71	1420
G	II	88	1232
H	II	94	1316
I	III	89	1157
J	III	94	4136
K	III	78	1560
L	III	71	1562
M	IV	98	1274
N	IV	58	2552
Total	—	1213	24,398

**Table 2:** Performance of the improved YOLOv11 model.

Networks	Precision	Recall	mAP@0.5	mAP@0.5:0.95	Parameters
YOLOv11s	0.861	0.893	0.879	0.540	9,401,275
Improved YOLOv11	0.915	0.948	0.925	0.615	19,250,030

**Table 3:** Performance comparison of different backbone networks.

Networks	Precision	Recall	mAP@0.5	mAP@0.5:0.95	Parameters
YOLOv11s	0.861	0.893	0.879	0.540	9,401,275
YOLOv11s-EfficientNet	0.858	0.875	0.872	0.520	8,950,210
YOLOv11s-ResNet50	0.882	0.920	0.905	0.585	33,200,550
YOLOv11s-DenseNet121	0.875	0.895	0.890	0.552	14,100,300
YOLOv11s-ShuffleNetV2	0.845	0.850	0.842	0.485	6,200,150
YOLOv11s-MobileNetV3	0.830	0.842	0.825	0.450	4,850,200
YOLOv11s-VGG11	0.885	0.915	0.902	0.570	15,100,050
YOLOv11s-VGG13	0.892	0.922	0.908	0.588	15,350,400
YOLOv11s-VGG16	0.895	0.935	0.912	0.595	21,600,800
YOLOv11s-VGG13bn	0.898	0.930	0.910	0.592	15,380,550

**Table 4:** Comparison of network performance before and after adding a small target detection head.

Networks	Precision	Recall	mAP@0.5	mAP@0.5:0.95	Parameters
YOLOv11s-VGG13bn	0.898	0.930	0.910	0.592	15,380,550
YOLOv11s-VGG13bn-P2	0.905	0.948	0.910	0.605	22,731,613

**Table 5:** Comparison of network performance before and after neck feature extraction improvement.

Networks	Precision	Recall	mAP@0.5	mAP@0.5:0.95	Parameters	Predicted Time (ms)
YOLOv11-VP	0.905	0.948	0.910	0.605	22,731,061	1.34
YOLOv11-VP-BiFPN	0.905	0.951	0.913	0.605	23,560,890	1.32
YOLOv11-VP-BiFPN- GhostConv	0.910	0.952	0.918	0.610	21,890,570	1.30
YOLOv11-VP-BiFPN- GhostConv-C3Ghost	0.915	0.953	0.925	0.615	19,250,030	1.27

**Table 6:** A comparison of different object detection methods.

Networks	Precision	Recall	mAP@0.5	mAP@0.5:0.95	Parameters
SSD	0.210	0.278	0.298	0.113	14,716,394
Faster RCNN	0.344	0.525	0.715	0.338	43,930,582
YOLOv3	0.845	0.845	0.850	0.483	8,699,906
RetinaNet	0.490	0.563	0.783	0.419	32,491,499
YOLOv5s	0.843	0.880	0.867	0.514	7,057,387
YOLOv8s	0.848	0.872	0.875	0.538	11,166,560
YOLOv10b	0.843	0.861	0.869	0.588	20,472,612
YOLOv11s	0.861	0.893	0.879	0.540	9,401,275
TOOD	0.872	0.885	0.884	0.562	28,150,420
RT-DETR	0.885	0.902	0.898	0.582	32,960,110
Improved YOLOv11	0.915	0.948	0.925	0.615	19,250,030

**Table 7:** Qualitative error patterns and failure mode analysis.

Error Category	Occurrence Rate (%)	Primary Manifestation
Boundary Mimicry	42.50%	False Positive
Reflection Occlusion	31.80%	False Negative
Geometric Distortion	25.70%	Localization Offset

**Table 8:** Cross-factory generalization results.

Training Source	Test Source	mAP@0.5	Precision	Volume RE (%)
Factories I, II, III	Factory IV	0.896	0.884	4.315

**Table 9:** Generalization performance on the HRIPCB dataset (Missing_hole).

Networks	Precision	Recall	mAP@0.5	mAP@0.5:0.95	Parameters
SSD	0.842	0.815	0.824	0.456	14,716,394
Faster R-CNN	0.915	0.892	0.908	0.512	43,930,582
YOLOv5s	0.928	0.931	0.935	0.584	7,057,387
YOLOv11s	0.942	0.938	0.949	0.612	9,401,275
TOOD	0.952	0.945	0.958	0.635	28,150,420
Improved YOLOv11	0.987	0.976	0.982	0.684	19,250,030

**Table 10:** Validation of AVC Algorithm against Certified Metrological Standards.

Sample ID	Metric	Certified Value	AVC Measured	RE (%)	SD
S1	Volume (mm^3^)	0.200	0.2069	3.45%	0.0042
	Surface Area (mm^2^)	2.600	2.4921	4.15%	0.0315
S2	Volume (mm^3^)	0.560	0.5812	3.79%	0.0085
	Surface Area (mm^2^)	4.320	4.1424	4.11%	0.0631

**Table 11:** Ablation study results for AVC algorithm components (Measured on Standard S1).

Case	Configuration	Volume RE (%)	Area RE (%)	Latency (ms)
1 (Baseline)	Cubic Fitting + N = 100 + Adaptive Map	3.45	4.15	51.4
2 (Fitting)	Linear Fitting (Degree 1)	8.42	9.10	42.1
3 (Fitting)	Quadratic Fitting (Degree 2)	5.21	6.05	48.6
4 (Step Size)	N = 20	7.15	7.82	31.2
5 (Step Size)	N = 50	4.62	5.34	44.5
6 (Mapping)	Fixed Rounding Mapping	4.12	5.35	50.2

**Table 12:** Performance comparison between AVC and classical Mesh-based Integration (S1 Standard).

Metric	Method	Certified Value	Measured Value	Relative Error (%)
Volume (mm^3^)	AVC	0.200	0.2069	3.45
	Mesh-based	0.200	0.2145	7.24
Area (mm^2^)	AVC	2.600	2.4921	4.15
	Mesh-based	2.600	2.7634	6.28

**Table 13:** Impact of localization offset on 3D measurement accuracy.

Localization Offset (Pixels)	IoU with Ground Truth	Surface Area RE (%)	Volume RE (%)
0 (Optimal)	1	5.236	3.964
±2	0.97	5.341	4.072
±5	0.92	5.612	4.285
±10	0.85	6.274	4.951
±15	0.78	7.915	6.533

**Table 14:** Breakdown of system-level latency and hardware sensitivity analysis.

Stage	Latency (RTX 3080)	Latency (Jetson AGX Orin)
$\mathrm{Acquisition}\;(t_{acq}$)	250.3 ms	360.0 ms
$\mathrm{Preprocessing}\;(t_{pre}$)	43.7 ms	95.5 ms
$\mathrm{Detection}\;(t_{det}$)	1.27 ms	8.15 ms
$\mathrm{Calculation}\;(t_{avc}$)	51.4 ms	173.5 ms
$\mathrm{Total}\;(T_{total}$)	346.67 ms	637.15 ms

## Data Availability

The data presented in this study are available on request from the corresponding author due to privacy.
